# Bee venom phospholipase A2 alleviates collagen-induced polyarthritis by inducing Foxp3^+^ regulatory T cell polarization in mice

**DOI:** 10.1038/s41598-021-82298-x

**Published:** 2021-02-10

**Authors:** Gwang-Muk Choi, Bombi Lee, Riwon Hong, Seon-Young Park, Da-Eun Cho, Mijung Yeom, Hi-Joon Park, Hyunsu Bae, Dae-Hyun Hahm

**Affiliations:** 1 Department of Biomedical Sciences, Graduate School, Kyung Hee University, Seoul, 02447 Republic of Korea; 2 Acupuncture and Meridian Science Research Center, Kyung Hee University, Seoul, 02447 Republic of Korea; 3 Department of Korean Medical Science, Graduate School, Kyung Hee University, Seoul, 02447 Republic of Korea; 4 Department of Physiology, College of Medicine, Kyung Hee University, Seoul, 02447 Republic of Korea; 5 BioNanocomposite Research Center, Kyung Hee University, Seoul, 02447 Republic of Korea

**Keywords:** Immunology, Health care, Pathogenesis, Rheumatology

## Abstract

The mechanism underlying bee venom (BV) therapy is still controversial, with opinions ranging from constituent-based pharmacological action to homeopathic-like activity. The purpose of this study was to examine whether BV phospholipase A2 (bvPLA2), an enzymatic component of BV, is a novel anti-inflammatory and anti-arthritic mediator capable of stimulating CD25^+^ Foxp3^+^ regulatory T cell (Treg) polarization in a mouse model of human rheumatoid arthritis (RA). An experimental model of RA was established in male DBA/1 mouse by 2-week-interval injections of 100 μg type II collagen emulsified in complete (first injection) or incomplete Freund’s adjuvant (second injection) at the base of the tail. During arthritis development, bvPLA2 (0.1, 0.5, 1.0 mg/kg) and/or Treg inhibitors such as anti-CD25 antibodies and peptide 60 (P60) were injected intraperitoneally for 5 weeks. Arthritic symptoms and the expansion of Tregs were then assessed by behavioral assessments, histological and micro-CT imaging, and flow cytometry. bvPLA2 injections significantly alleviated arthritic behaviors such as squeaking and joint swelling, consistent with changes seen on both histological and micro-CT images. The anti-arthritic effects of bvPLA2 were blocked by intraperitoneal injections of 0.25 mg/kg anti-CD25 antibody and 10 μg/kg P60, as determined by behavioral assessments. Flow cytometric analysis of dendritic cells, B cells, and major T cell subsets from spleens revealed a significant depletion of Tregs following anti-CD25 antibody, but not P60, treatment. bvPLA2 treatment exerted significant anti-inflammatory and anti-arthritic activities in a mouse model of RA via the induction of Tregs.

## Introduction

Honeybee venom is a bitter, colorless liquid containing a complex mixture of toxic peptides, enzymes, and bioactive amines secreted from the venom sacs of honeybees. Bee sting therapy has been used in traditional Chinese medicine for over 3000 years as a treatment for chronic pain and inflammation. This therapy has been further adapted in the form of bee venom (BV) acupuncture, a novel type of acupuncture in which a small volume of purified and diluted BV is injected into specific skin points to reinforce the stimulation of acupuncture points^1,2^.

In recent years, BV acupuncture has been proposed as a potential treatment for a variety of neurological diseases, including peripheral neuropathies, stroke, Parkinson’s disease, aging-associated memory, and learning deficits, as well as pain and inflammatory diseases such as chronic musculoskeletal pain and osteoarthritis^1–8^. Previous studies have found that three main peptides, such as melittin, apamin, and mast cell degranulating peptide, serve as key mediators of its medicinal activity. For example, melittin, the most abundant component of BV, is responsible for the homeopathy-like systemic effects of BV therapy, and it serves as the major pain-inducing component of bee stings. However, the concentration-dependent bidirectional effects of BV, that is, its non-specific inhibition of receptor-mediated cell signaling and destruction of nearby cells, combined with its anti-inflammatory and antinociceptive properties, have not been clearly elucidated.

Recently, secretory PLA2, an important component of BV shown to trigger allergy and anaphylactic shock when injected at high concentrations, was proposed as a possible therapeutic compound due to its potent anti-nociceptive and anti-inflammatory effects at lower concentrations^9–13^. PLA2 hydrolyzes the ester bond at the sn-2 position of membrane phospholipids to liberate lysophosphatidic acid and free fatty acids including arachidonic acid, which become inflammatory and cytotoxic on various types of cells^14,15^. In several studies, the extracellular forms of PLA2, including bvPLA2, were shown to be effective for alleviating neuroinflammation in Alzheimer’s disease and Parkinson’s disease as well as allergic inflammation in atopic dermatitis and asthma, and those anti-inflammatory effects seemed be associated with modulation of regulatory T cell (Treg) differentiation^9–13^. Although the mechanism underlying bvPLA2-induced Treg polarization has not been clearly identified, bvPLA2 was shown to bind to CD206 on dendritic cells, resulting in the expression of Foxp3 in the neighboring naïve T cells and their polarization into Tregs^10,12,13,16^.

In the mammalian immune system, Tregs play an important role in modulating aberrant and exaggerated immune responses after elimination of the invading pathogen by boosting the innate immune response. Tregs are thus important for the maintenance of immune self-tolerance and homeostasis via downregulation of effector T cells^17^. In this regard, any therapies capable of inducing the differentiation and expansion of Tregs may be effective for the treatment of autoimmune diseases such as rheumatoid arthritis (RA), psoriasis, type I diabetes, myasthenia gravis, multiple sclerosis, and systemic lupus erythematosus^18–26^.

One of the most successful uses of traditional bee sting therapy to date has been for the treatment of RA. T cell selection and antigen presentation are crucial steps in the initial stage of RA pathophysiology. Attenuation of these processes by BV is thought to be mediated by the stimulation of Treg development and expansion. It was previously reported that depletion of CD4^+^ CD25^+^ Tregs exacerbated arthritic symptoms in RA-like rodent models, while adoptive transfer of Tregs significantly attenuated RA symptoms^25,27,28^. In the present study, intraperitoneal injection of bvPLA2 is proposed as a novel therapeutic to treat systemic inflammatory symptoms of collagen-induced arthritis (CIA) via stimulation of CD4^+^ CD25^+^ Treg polarization. To the best of our knowledge, this is the first study to examine the association of Treg polarization with the anti-RA activity of bvPLA2 in a CIA mouse model of human RA. To verify this, the inhibitory effects of bvPLA2 on arthritic symptoms were reassessed after CD25^+^ Foxp3^+^ Tregs were completely depleted by treatment with an anti-CD25 antibody (Ab) in CIA mice, and the bvPLA2-induced changes in major T cell subsets including CD25^+^ Foxp3^+^ Tregs were analyzed by flow cytometry.

## Results

### Behavioral assessment of the anti-arthritic activity of bvPLA2

In this study, the CIA mouse model was used to examine the anti-arthritic activity of bvPLA2. Body weight, the squeaking score, paw thickness, and the arthritis index were used to assess the clinical symptoms of arthritis (Fig. 2A–D). Body weight began to decrease in the untreated arthritis group (CIA) approximately 2 weeks after the second immunization. Body weight loss in the CIA group was significantly inhibited by bvPLA2 treatment in a dose-dependent manner. The weight restoration in the 1.0 mg/kg PLA2-treated arthritis group was closest to that in the 2 mg/kg MTX-treated group used as the positive control. Squeaking score, which was used as an indicator of arthritic ankle pain, began to increase after the second immunization on day 14 and reached a maximum on day 30 in the CIA group. In contrast, the squeaking score was significantly alleviated by *i.p.* injection of bvPLA2, which was started on day 18, in a dose-dependent manner. Paw thickness, an indicator of arthritic edema, and the arthritis index, a comprehensive marker of arthritic pain and inflammation, also exhibited behavioral patterns similar to those of the squeaking score in the bvPLA2-treated groups.

### Histological and μCT imaging assessments of knee joints confirming the anti-arthritic activity of bvPLA2

The apparent arthritic severity and histological sections of the knee joints stained with H&E were characterized with regard to the degrees of ankle deformity, cartilage erosion, and synovial hyperplasia of the affected knee joints to further evaluate the anti-arthritic activity of bvPLA2. Pathological abnormalities of arthritic joints were scored in seven specimens based on a combination of photos and histological images from each experimental group. All scoring was performed by three independent pathologists who were blinded to the treatments given to the mice. The scores were based primarily on the growth of the cartilage–pannus junction and the thickness of the synovial membrane (small and large blue squares in Fig. 3), the number of infiltrated immune cells (yellow arrows in black squares), and the degree of inflammation seen in the hind paws (A’ to F’). Knee joint sections from arthritic mice in the CIA group (Fig. 3B) exhibited increased pannus formation, joint space narrowing, and cartilage loss, thicker synovial tissues, and increased immune cell infiltration into the inflamed synovial tissues compared with naïve mice in the NOR group (Fig. 3A). bvPLA2 treatment effectively alleviated the histological signs of collagen-induced abnormalities in the knee joints in a dose-dependent manner (Fig. 3B–E). The mean severity of the histological findings was quantitatively summarized as an inflammation score in the bar graph in Fig. 3G. Photographic and histological observations of 1.0 mg/kg bvPLA2-treated mouse knee joints were similar to those receiving MTX treatment.

To confirm the extent of bone erosion, 3D images of knee joints and 2D images of trabecular and cortical bones were taken from NOR, CIA, and bvPLA2-treated mice during in vivo μCT scanning. Severe erosion of bone and cartilage in the knee joint, loss of bone microarchitecture, and decreased cortical thickness and BMD were observed in arthritic mice from the CIA group, compared with the normal mice (Fig. 4A–F). The strong concordance between behavioral and histological analyses indicates that bvPLA2 treatment significantly inhibited the bone and cartilage destruction observed in the CIA group.

### Effect of selective Treg depletion by anti-CD25 Ab on the anti-arthritic activity of bvPLA2

It was previously reported that bvPLA2 had anti-inflammatory and analgesic effects in rodent models of Parkinson’s disease, asthma, neuropathic pain, and drug-induced liver and kidney injuries via modulation of Foxp3^+^ regulatory T cells^12–16^. We thus examined whether the anti-arthritic effects of bvPLA2 are attributed to its effects on Treg polarization. Depletion of Treg populations using a rat anti-mouse CD25 Ab was performed, followed by injection of 0.5 mg/kg bvPLA2 in CIA mice. Anti-CD25 Ab injections were initiated 4 days prior to bvPLA2 treatment to ensure sufficient depletion of Treg populations. As shown in Fig. 5, the anti-arthritic effects of bvPLA2 were noticeably lost by 50 days post-immunization with CII + CFA, as evidenced by changes in body weight, squeaking score, paw volume, and arthritis index. The injection of normal rat Ab, used as the vehicle control, did not affect bvPLA2-mediated changes in arthritic behaviors.

As an alternative strategy to control Tregs in vivo, we also used P60, a synthetic peptide that binds to FOXP3, a factor required for Treg development and function, thus inhibiting their function to protect against autoimmune diseases and prevent rejection of allogeneic transplants. As in the anti-CD25 Ab-treated mice, P60 significantly attenuated the anti-arthritic activity of bvPLA2, with respect to body weight and arthritic behaviors, when simultaneously injected with bvPLA2 into the CIA mice (Fig. 5). In the squeaking score evaluation, the inhibitory effect of P60 on bvPLA2 was greater than the effect of anti-CD25 Ab (Fig. 5B). Scrambled peptide, used as a negative control for P60, did not affect bvPLA2 activity.

### Recovery profile of T cell subsets in the spleen of CIA mice following bvPLA2 and P60 treatments

To examine changes in Treg populations following simultaneous treatment of bvPLA2 with one of its inhibitors, such as anti-CD25 Ab and P60, the populations of CD11c^+^ dendritic cells (DCs), CD19^+^ B cells, CD8^+^ cytotoxic T cells, CD4^+^ helper T cells, and CD4^+^ CD25^+^ Foxp3^+^ Tregs were analyzed in the spleens from each group using a flow cytometer. As shown in Fig. 6., the proportion of CD8^+^ and CD4^+^ T cells were significantly reduced in the spleens of CIA mice, as compared with NOR mice, whereas both DCs and CD19^+^ B cells remained largely unaffected. The proportions of CD4^+^ CD25^+^ Foxp3^+^ Tregs and CD4^+^ helper T cells were not affected in any of the CIA groups compared with the NOR group. The injection of bvPLA2 into CIA mice increased the proportions of all immune cells tested in this study despite having no significant effects on CD8^+^ and CD4^+^ T cell numbers. Among the cell types assessed, the CD4^+^ CD25^+^ Foxp3^+^ Treg population among CD4^+^ T cells showed the most remarkable increase (Fig. 6E). However, bvPLA2 injection did not affect the proportion of DCs, B cells, T cells, or Tregs relative to the NOR group (data not shown). Pretreatment with anti-CD25 Ab completely blocked the bvPLA2-induced effects on immune cell populations such as DCs, B cells, and CD4^+^ CD25^+^ Foxp3^+^ Tregs. Interestingly, bvPLA2-mediated expansion of the CD4^+^ CD25^+^ Foxp3^+^ Treg population among CD4^+^ helper T cells was inhibited most dramatically following treatment with anti-CD25 Ab. This inhibitory effect was not observed following treatment with another Treg inhibitor, P60.

## Discussion

Phospholipase A2 (PLA2) is highly conserved across a wide variety of organisms including bacteria, fungi, plants, insects, reptiles, and mammals, and plays specialized roles in host defense, tissue differentiation, and membrane remodeling^16^. Under pathological conditions, arachidonic acid, released from membrane phospholipids by PLA2, is further metabolized into a variety of biologically active eicosanoids including prostaglandins and leukotrienes, which are primarily inflammatory in nature. In patients suffering from RA and other immune-mediated inflammatory diseases, the excessive consumption of arachidonic acids as dietary supplements can exacerbate their symptoms, presumably due to their conversion into inflammatory intermediates^29^.

As in the venom from multiple species, bvPLA2 acts as a primary allergen sensed by the innate immune system and induces a type 2 immune response after injection into mammalian tissues^30^. Although the detailed mechanism by which how PLA2 activates Th2 and IgE responses as an exogenous protein allergen remains unclear, it is thought that PLA2s are detected via immune sensing of the enzyme itself or as secondary effects of their downstream enzyme activities, as seen in protease treatment of parasitic worms such as helminth^30^.

Secretary forms of PLA2 bind to specific membrane receptors in skeletal muscles (M-type receptors) and rodent brains (N-type receptors), where they act as a non-specific ligand of their various downstream enzymatic activities^16^. While mammalian pancreatic or non-pancreatic sPLA2s can preferably bind to M-type receptor, a low-molecular weight sPLA2 from BV appears to act as a ligand through the high-affinity binding to N-type receptor.

In contrast to previous studies, Prof. Bae’s group recently reported that bvPLA2 exhibited a potent neuroprotective effect in rodent models of Alzheimer’s and Parkinson’s diseases via the modulation of Foxp3-expressing CD4^+^ Treg expansion, and that the therapeutic action of BV against various neuroinflammatory diseases might be attributed to the anti-inflammatory effect of Foxp3^+^ Tregs activated by bvPLA2. The authors also showed that bvPLA2 treatment was highly effective on drug-induced liver and kidney injuries, as well as acute pain and allergic inflammation in appropriate animal models^9–12^. It was also suggested that bvPLA2 binds to CD206 on dendritic cells, which in turn activates PGE2 signaling, resulting in the expansion of Tregs^13^.

The CD206 mannose receptor on dendritic cells and E2/EP2 signaling in CD4^+^ T cells seem to be associated with the crosstalk among bvPLA2, dendritic cells, and CD4^+^ T cells. However, it is still not clear whether bvPLA2 binds directly to one or more receptors on dendritic cells to initiate Treg polarization, or whether its enzymatic byproducts interact with those receptors. If bvPLA2 does bind directly to one or more receptors, the binding moieties have not been identified. Furthermore, the mechanism by which PLA2-activated dendritic cells stimulate the differentiation of naïve T cells into Tregs is also unknown.

Quantitative and/or functional abnormalities of Tregs play a vital role in the natural development of autoimmune diseases including RA, as these cells help modulate immune responses to environmental pathogens and are therefore essential for establishing self-tolerance^31^. It can therefore be hypothesized that PLA2 provides an effective treatment for inflammatory autoimmune diseases such as RA despite its known allergenic properties, mediated by the conversion of naïve CD4^+^ T cell into Foxp3^+^ Tregs.

In the present study, the anti-RA effects of bvPLA2 were attributed to the bvPLA2-induced quantitative expansion of CD25^+^ Foxp3^+^ Tregs in the CIA mouse model. To neutralize the Treg-mediated anti-arthritic activity of bvPLA2, two different inhibition strategies were utilized, both quantitative depletion of Tregs using an anti-CD25 Ab and their functional inactivation using a peptide inhibitor to ablate FOXP3 in Tregs^10–12,32,33^. The P60 inhibitor works by entering the cell, where it suppresses Foxp3 nuclear translocation, thereby reducing its ability to inhibit NF-κB and NFAT, resulting in the inhibition of Treg differentiation or function^33–35^. In 2010, Lasarte’s research group isolated P60, a 15-mer synthetic peptide, from a phage-displayed random peptide library to bind to FOXP3 and inhibit its activity as an intracellular transcription factor. They also found that P60 penetrated the cell membrane and inhibited FOXP3 nuclear translocation within the cells in vitro and in vivo^33^. Repeated injections of the anti-CD25 Ab not only inhibited the anti-arthritic effects of bvPLA2, as evidenced by multiple arthritic behaviors (Fig. 5), but also neutralized the bvPLA2-induced increase in the proportion of CD25^+^ FoxP3^+^ Tregs (Fig. 6E). In contrast, even though P60 exhibited comparable inhibitory effects on CIA-associated pain and edema in mice treated with anti-CD25 Ab, it did not affect the proportion of Tregs in lymph nodes (Fig. 6E). It can be assumed that P60, after entering the cells, interacted with FoxP3 in the cytoplasm after FoxP3 proteins, fully expressed in CD4 + T cells by the treatment of bvPLA2. P60 in the cell cytoplasm can affect only the activity of Foxp3 but not its expression levels stimulated by bvPLA2 treatment. It seems to be why the P60 treatment did not decrease the population size of Foxp3^+^CD25^+^ Tregs although it clearly showed the inhibitory effect on the Tregs-mediated anti-arthritic behaviors in bvPLA2-treated CIA mice. Scrambled peptides, composed of the same number of amino acids as those in P60 but in a random order, did not alter either the arthritic behaviors or Treg levels in lymph nodes.

In the present study, CIA mice showed histological features similar to those of human RA patients^36,37^. These features, including joint deformity, cartilage loss, and bone erosion, were further confirmed by μCT analyses. As another traditional marker of arthritic erosion, the BMD measured by μCT also enabled assessment of bone loss in CIA mice^38^. Using μCT, the PLA2-mediated increase in Treg expansion was found to be effective for improving bone metabolism and attenuating inflammatory responses in the affected joints and surrounding tissues in RA.

The anti-arthritic efficacy of BV therapy was shown to be directly attributable to bvPLA2, one of the main components of BV, and this effect was closely associated with expansion of CD25^+^ Foxp3^+^ Tregs in the mouse model of RA. Further investigations will be necessary to understand the full cascade of events mediating these effects, including targeting of Tregs by bvPLAS2, and the interaction and binding mechanisms between the moiety of bvPLA2 and antigen-presenting cells.

## Methods

### Animals

Six-week-old male DBA/1 mice (19.5 ± 0.5) were obtained from Raon Bio (Youngin, South Korea). The mice were kept under environmentally controlled conditions (temperature: 22 ± 2 °C; humidity: 55 ± 16%) and a 12/12 h light/dark cycle (lights on at 08:00, off at 20:00) and were acclimated to these conditions before the experiment for at least 1 week. The maximum caging density was five mice from the same litter and sex starting from weaning. As bedding, autoclaved wood fiber soft chips (Lignocel® 3/4-S; J. Rettenmaier & Söhne GmbH + Co KG, Rosenberg, Baden-Württemberg, Germany) were provided. Mice were fed a standardized mouse diet (+ 40 RMM; Safe, Augy, Bourgogne-Franche-Comté, France) and provided drinking water ad libitum. All materials, including individually ventilated cages, lids, feeders, bottles, bedding, and water were autoclaved before use. Efforts had been always devoted to minimize the number of animals used per experiment or test and their potential suffering. All animal care and experimental procedures were conducted in accordance with the National Institute of Health Guide for the Care and Use of Laboratory Animals and were approved by the Kyung Hee University Institutional Animal Care and Use Committee (KHUASP(SE)-19-052).

### Reagents

PLA2 was purchased form Sigma-Aldrich Chemical Co. (St. Louis, MO, USA). Chicken type II collagen (CII), complete Freund’s adjuvant (CFA), and incomplete Freund’s adjuvant (IFA) were purchased form Chondrex Inc. (Redmond, WA, USA). Peptide 60 (P60; RDFQSFRKMWPFFAM) and its scrambled peptide were synthesized from ChemPeptide Limited (Shanghai, China). The rat anti-mouse CD25 Ab (clone PC61), derived from hybridomas obtained from the American Type Culture Collection (Manassas, VA, USA), was kindly provided by Prof. Bae’s laboratory at Kyung Hee University^13^.

### Collagen-induced arthritis (CIA)

**Figure 1:**
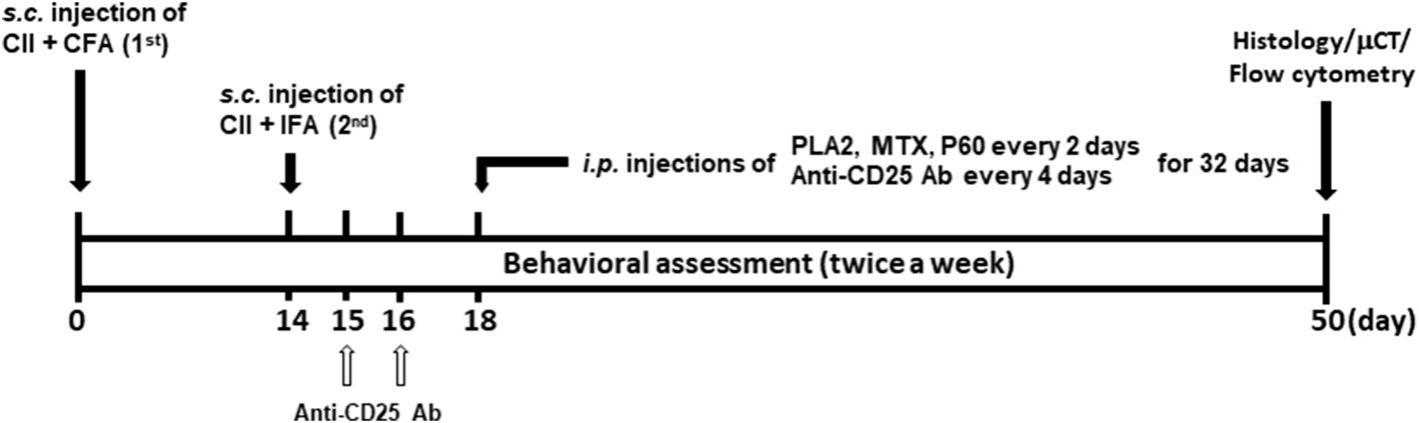
Schematic drawing of the experimental schedules. RA-like symptoms were induced by 2-week-interval double injections of 100 µg chicken type II collagen (CII) emulsified in complete Freund’s adjuvant (CFA, first injection) or incomplete Freund’s adjuvant (IFA, second injection) at the base of the tail. An anti-CD25 antibody (Ab) was injected *i.p.* twice (on days 15 and 16) prior to the onset of arthritic symptoms (onset stage), followed by an additional eight treatments over the course of 4 weeks. Other drugs such as PLA2, P60 (Treg inhibitor), and MTX (positive control) were injected every 2 days for 32 days after disease onset. PLA2: bee venom phospholipase A2; P60: peptide P60; MTX: methotrexate; anti-CD25 Ab: anti-mouse CD25 rat antibody (IgG).

After a 1-week-adaptation period, 50 μL stable emulsion consisting of 100 µg chicken CII and CFA containing 200 µg *Mycobacterium tuberculosis* H37Ra in mineral oil was injected subcutaneously into the base of the tails of DBA/1 mice. Two weeks after the first injection, the mice were treated via a second injection of 50 μL emulsion consisting of 100 µg chicken CII and IFA. The day of the first immunization injection was designated as day 0. The animals were included in the study if they underwent noticeable edema on both ankle joints on 3rd day after second immunization, defined by the increase of 10% or greater edema measured with water-displacement plethysmometer (Ugo-Basil Biological Research Apparatus Co., Comerio-Varese, Italy). If the animal died prematurely, preventing the collection of behavioral and histological data. Methotrexate (MTX) and P60 were injected i.p. with bvPLA2 every 2 days starting on day 18 until day 50, and anti-CD25 Ab were injected i.p. on days 15 and 16, and every 4 days thereafter starting on day 18 until day 50. Mice were euthanized for end-point sampling by use of CO2 inhalation and cervical dislocation. Euthanized mice were dissected, and spleens and knee joints were removed to prepare a single cell suspension from spleen and paraffin block embedding, respectively, as described previously^12^. An overview of the experimental schedule is depicted in Fig. 1.

### Experimental groups

Two sets of animal studies were performed. In the first study, we examined the anti-arthritic effects of bvPLA2 (Exp. 1), and in the second study, we investigated the inhibitory effects of bvPLA2 by depletion of Treg (Exp. 2) in the CIA mouse model. In Exp. 1, mice were randomly divided into six groups: the untreated naïve (NOR, *n* = 7), CIA (*n* = 7), 0.1 mg/kg PLA2-treated arthritis (*n* = 7), 0.5 mg/kg PLA2-treated arthritis (*n* = 7), 1.0 mg/kg PLA2-treated arthritis (*n* = 7), and 2 mg/kg MTX-treated arthritis (MTX, *n* = 7) groups. In Exp. 2, mice were divided into seven groups: untreated naïve (NOR, *n* = 6), CIA (*n* = 6), 0.5 mg/kg PLA2-treated arthritis (*n* = 6), 0.5 mg/kg PLA2- and 0.25 mg/kg rat anti-mouse CD25 Ab-treated arthritis (anti-CD25 Ab, *n* = 6), 0.5 mg/kg PLA2- and 10 μg/kg P60-treated arthritis (P60, *n* = 6), 0.5 mg/kg PLA2- and 0.25 mg/kg normal rat Ab-treated arthritis (*n* = 6), and 0.5 mg/kg PLA2- and 10 μg/kg scrambled peptide-treated arthritis (*n* = 6) group.

### Behavioral assessment

To evaluate the clinical severity of arthritis, body weight and three behaviors, including squeaking score, paw swelling (thickness), and arthritis index, were assessed twice weekly beginning with the first immunization and continuing until day 50. Body weight was measured using a digital balance (Mettler-Toledo Inc., Columbus, OH, USA). Ankle pain was evaluated on a scale measuring squeaking to assess nociception and hyperalgesia. Squeaking included any vocalization evoked by forced ankle flexion and extension^2^. The flexion–extension procedure was repeated 10 times in total, once every 5 s, and a grade of 0 (no vocalization) or 1 (vocalization) was given for each hind limb of the mouse. The total number of vocalizations, as detected by an observer blinded to the animal groups, was calculated as the squeaking score. Paw thickness (swelling) was determined by measuring the ankle thickness of the arthritic hindlimb. The thickness of the most swollen part of the ankle was measured using a digital caliper (Mitutoyo, Sakado, Japan). The arthritis index was assessed by grading the apparent arthritic severity in all joints of each limb using a four-point scale per limb; the maximum score was 16 for each mouse, where 0 = no erythema or swelling of any joint in one limb; 1 = erythema or swelling of at least one joint per limb; 2 = erythema or swelling of fewer than three joints per limb; 3 = erythema or swelling of all joints in one limb; and 4 = ankylosis and deformity of all joints in one limb. Due to overt behavioral activity, the experimenter could not be blinded to whether the animal was injected with drugs or with saline.

### Histology

Hematoxylin and eosin (H&E) staining of knee joint sections was performed to evaluate synovial pathology, deformities of knee ankles, and immune cell infiltration in the affected joints. All mice were euthanized on day 50 after the first immunization with CII + CFA. Knee joints were dissected, fixed in 10% neutral buffered formalin (Sigma-Aldrich, St. Louis, MO, USA) for 24 h, and decalcified in Calci-Clear Rapid solution (National Diagnostics, Atlanta, GA, USA) until the bones were pliable. The joints were then dehydrated through a graded ethanol series, cleared in xylene, and processed for embedding in paraffin wax using routine protocols. Coronal sections (8 μm) were cut through the knee joint using a rotary microtome (Finesse 325, Thermo Shandon Inc., Pittsburgh, PA, USA) and stained with H&E for routine histological evaluations. The samples were examined with a semi-motorized fluorescence microscope (Olympus BX53, Olympus Co., Japan).

### Micro-CT (μCT)

Knee joint samples were dissected and fixed with a 10% formalin solution to prepare them for μCT. 3D and 2D imaging analyses of the joints were performed using an in vivo μCT system (NFR Polaris-G90; NanoFocus Ray Co., JeonJu, South Korea). The following settings were employed: X-ray voltage of 55 kV, X-ray current of 105 mA, X-ray spot size of 8 mm, and exposure time of 80 ms for each 180° rotational step. Reconstruction of 3D images of the knee joints and 2D images of the trabecular bone (in a sagittal section at the top of the tibia) and of the cortical bone (in a horizontal section in the middle of the tibia) was performed using the Data viewer, CTVox, and CTAn software packages (SkyScan, Kontich, Belgium) on 500 slices. The bone mineral density (BMD) of each reconstructed knee joint was measured at 1 mm from the top of the tibia after setting the region of interest.

### Cell isolation and flow cytometry

Spleens were chopped into small pieces and mechanically dissociated using a 40 µm nylon mesh strainer (BD Falcon, Schaffhausen, Switzerland). Red blood cells were lysed for 5 min in 1X BD Pharm Lyse buffer (BD Pharmingen Inc./BD Biosciences, San Diego, CA, USA), after which the remaining cells were stained with fluorescently tagged Ab. All data were collected using the FACSCalibur cytometric system (Becton Dickinson, San Jose, CA, USA) and analyzed by FlowJo software (TreeStar, Inc., San Carols, CA, USA). The following fluorophore-labeled Ab were used: CD8-APC, Foxp3-PE cy5, and CD19-PE from e-Bioscience, Inc.; CD11c-APC cy7 and CD25-FITC from BioLegend (San Diego, CA, USA); and CD4-PE cy7 from Invitrogen (Carlsbad, CA, USA).

### Statistical analysis

**Figure 2:**
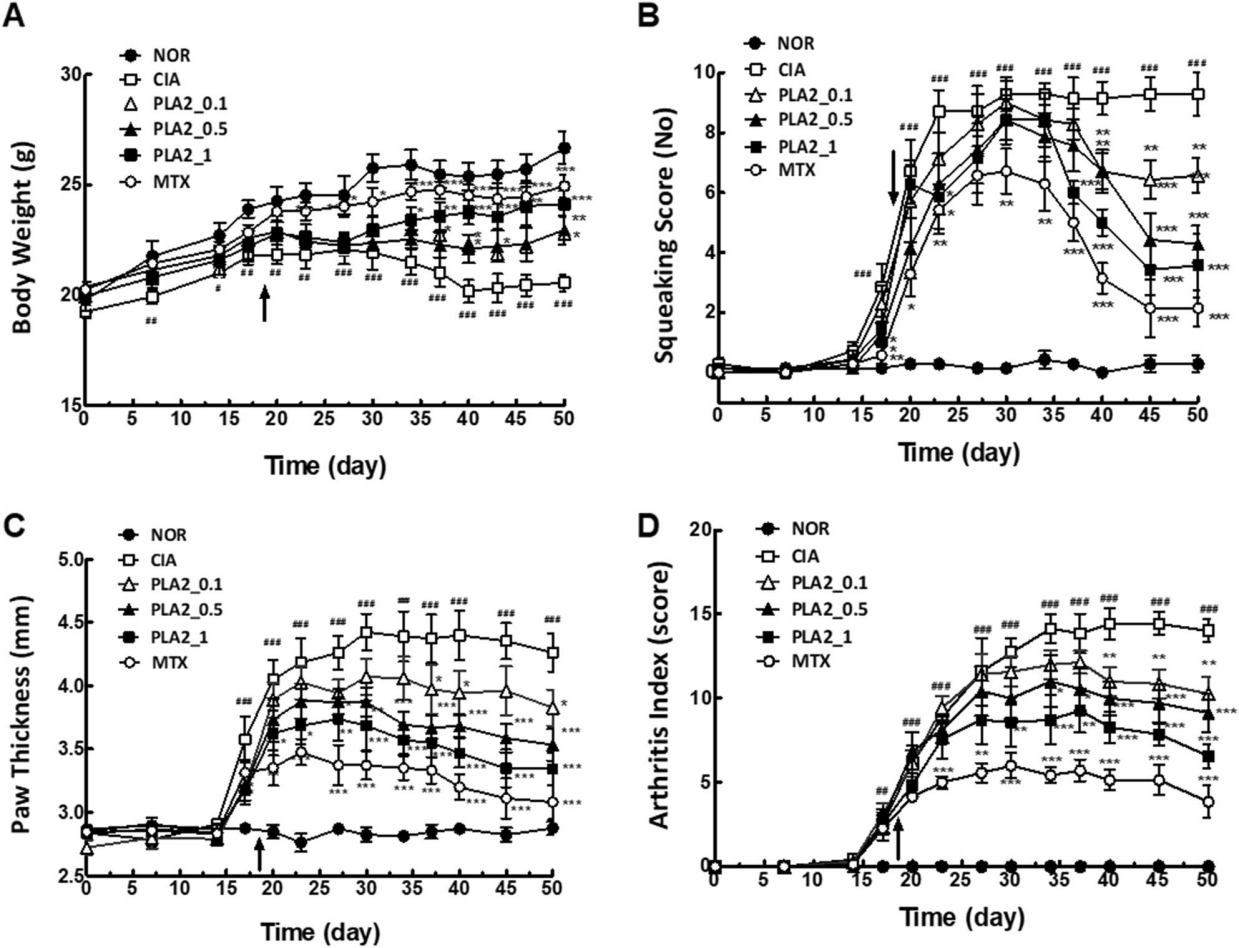
Behavioral assessment of the anti-arthritic activity of PLA2 in DBA/1 mice with CIA in terms of (**A**) body weight, (**B**) the squeaking score, (**C**) paw thickness, and (**D**) the arthritis index. The arrow indicates the starting day of *i.p.* administration of bvPLA2, MTX, and P60. NOR: untreated naïve group (*n* = 7); CIA: CIA group (*n* = 7); PLA2_0.1: 0.1 mg/kg PLA2-treated arthritis group (*n* = 7); PLA2_0.5: 0.5 mg/kg PLA2-treated arthritis group (*n* = 7); PLA2_1: 1.0 mg/kg PLA2-treated arthritis group (*n* = 7); MTX: 2 mg/kg methotrexate-treated arthritis group (*n* = 7). PLA2: bee venom phospholipase A2. Data for body weight, squeaking score, paw volume, and arthritis index were analyzed using one-way ANOVA, followed by Bonferroni’s post-hoc test. In all analyses, statistical differences were considered significant at* p* < 0.05. *p < 0.05, **p < 0.01, ***p < 0.001 versus the CIA group, and ^#^*p* < 0.05, ^##^*p* < 0.01, ^###^*p* < 0.001 versus the NOR group.

**Figure 3:**
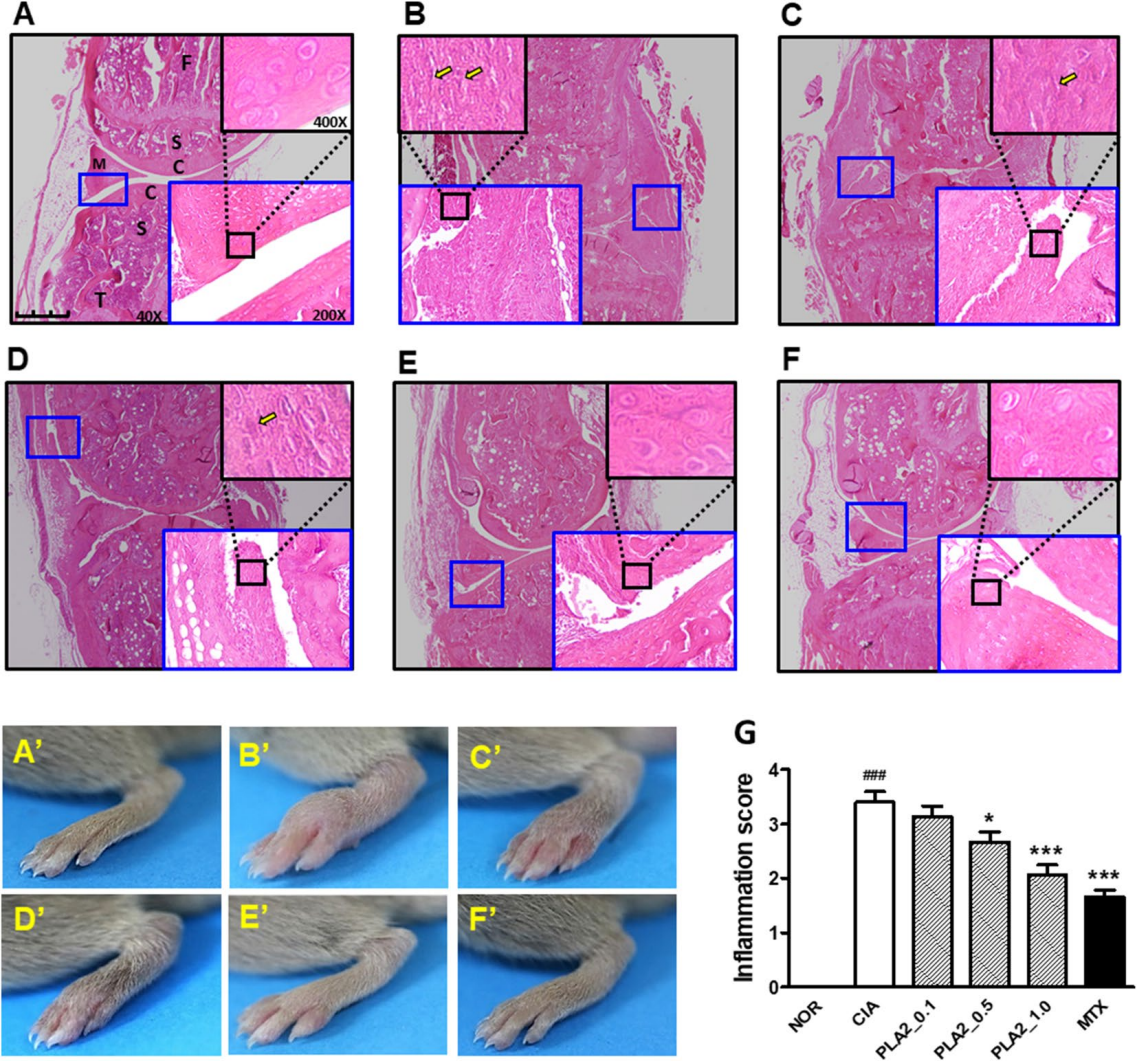
H&E-stained histological images of knee joints and hindlimb photographs of the NOR (**A**,**A’**), CIA (**B**,**B’**), PLA2_0.1 (**C**,**C’**), PLA2_0.5 (**D**,**D’**), PLA2_1 (**E**,**E’**), and MTX (**F**,**F’**) groups, and a bar graph (**G**) indicating the arthritic severity based on histological images and hindlimb photos. Tissues were stained with hematoxylin and eosin (H&E, × 40). Scale bar = 2 mm. The extent of inflammation in the histological images was scored on a scale of 0–4 based on apparent redness and edema in hindlimb feet, as well as hypertrophy of the synovium tissues (large blue line squares) and immune cell infiltration into the transformed synovium tissues (yellow arrows in black line squares). Scoring was performed by three pathologists blinded to the animal groups, where 0 = normal (no inflammation), 1 = minimal inflammation, 2 = mild inflammation, 3 = moderate inflammation, and 4 = severe inflammation. Enlarged histological figures in black squares in the upper right corners (400 ×) indicate inflammatory cell infiltrates (yellow arrows in **B**,**C**,**D**). Small blue squares in H&E-stained images are magnified in the large blue squares at the lower right corner (× 200). C: cartilage; S: subchondral bone; F: femur; T: tibia; M: meniscus. One-way ANOVA followed by Newman–Keuls post hoc test was used for comparison of inflammation score among the groups. In all analyses, statistical differences were considered significant at* p* < 0.05. ^###^*p* < 0.001 versus the NOR group, and *p < 0.05, ***p < 0.001 versus the CIA group.

**Figure 4:**
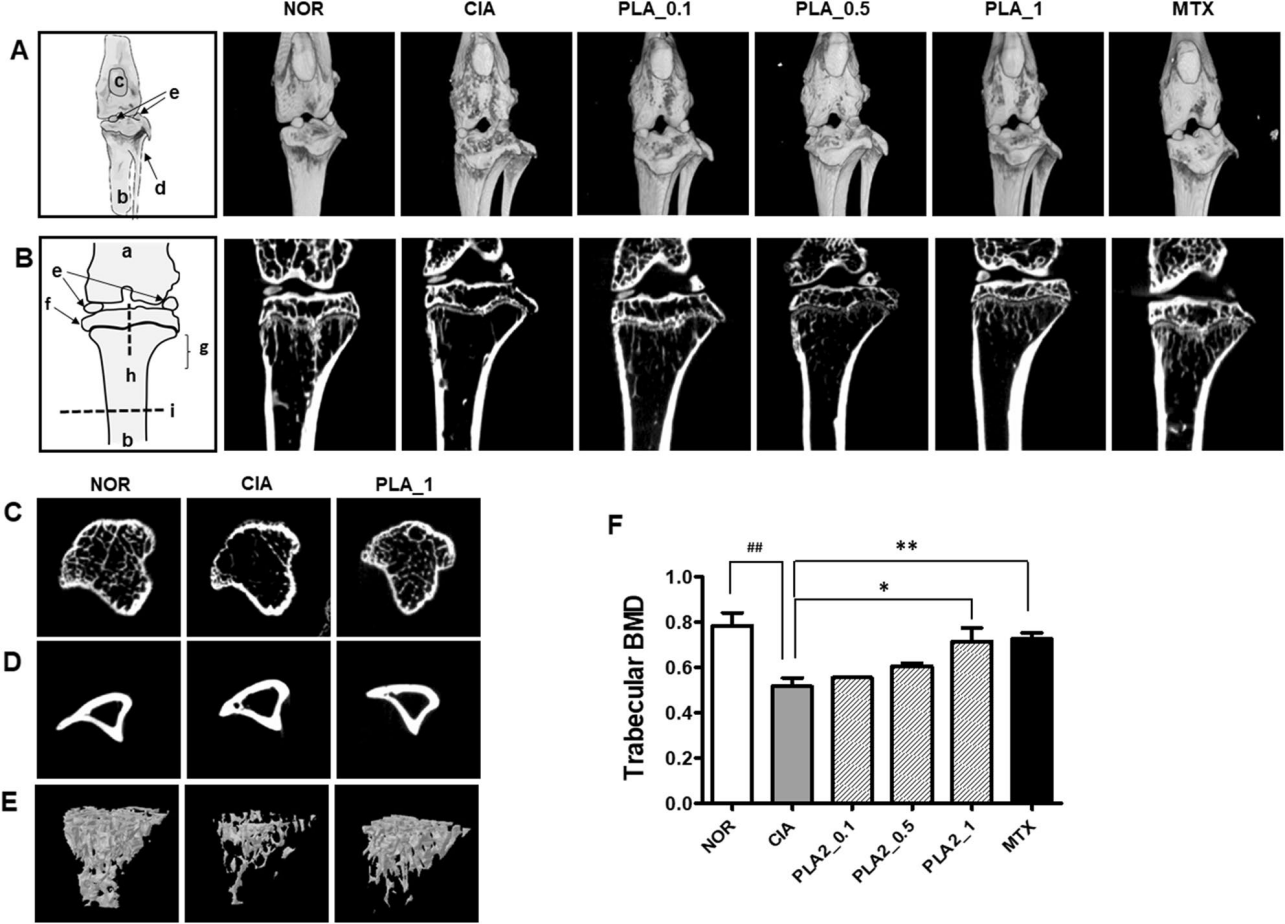
Representative 3D images of the entire knee joint (**A**), 2D images of the sagittal section at the tops of the femur and tibia (**B**), 2D images of the horizontal sections of cancellous (**C**) and cortical (**D**) bones in the middle of the tibia, 3D images reconstituted from 100 sagittal sections of cancellous bone at the top of the tibia (**E**), and a bar graph (**F**) showing the bone mineral density (BMD) of the knee joints to estimate joint corrosion and cartilage loss in arthritic DBA1/J mice. Dotted lines in the schematic diagram indicate the direction of sagittal sections (h) producing 3D images in (**E**). Horizontal sections (i) producing 2D images of (**C**) and (**D**). a: femur; b: tibia; c: patella; d: fibula; e: sesamoid bone; f: epiphysis; g: metaphysis; bvPLA2: bee venom phospholipase A2; MTX: methotrexate. One-way ANOVA followed by Newman–Keuls post hoc test was used for comparison of bone mineral density (BMD) among the groups. In all analyses, statistical differences were considered significant at* p* < 0.05. ^##^*p* < 0.01 versus the NOR group, and *p < 0.05, **p < 0.01 versus the CIA group.

**Figure 5:**
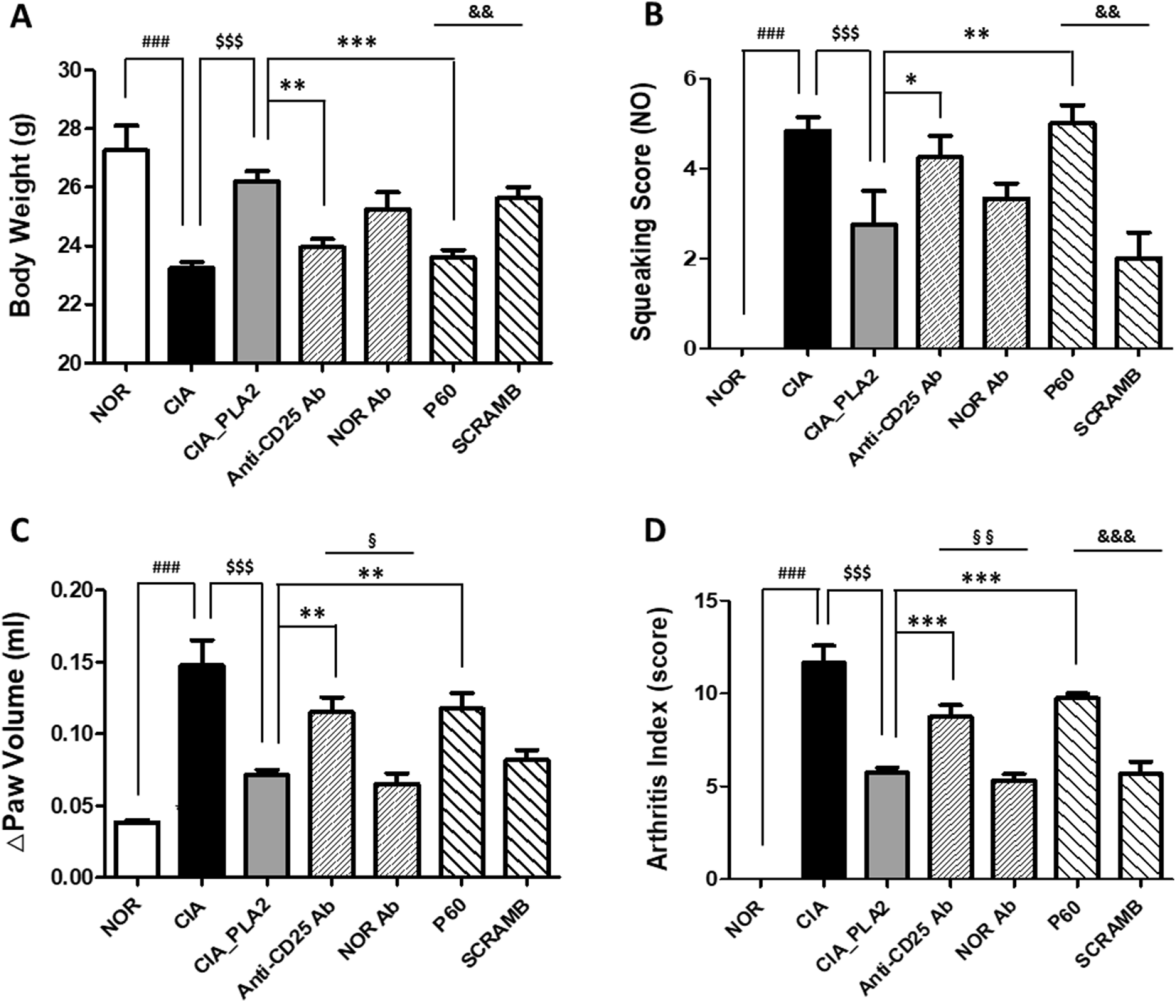
Effect of Treg depletion, via anti-mouse CD25 Ab or P60 treatment, on the anti-arthritic activity of PLA2 in DBA/1 mice with CIA. Fifty days after the first immunization injection, mice were analyzed based on body weight (**A**), the squeaking score (**B**), change (Δ) in paw volume (**C**), and the arthritis index (**D**). Clinical evaluations of mouse limbs were performed on day 50. NOR: untreated naïve group (*n* = 6); CIA: CIA group (*n* = 6); CIA_PLA2: 0.5 mg/kg bvPLA2-treated arthritis group (*n* = 6); anti-CD25 Ab: 0.5 mg/kg bvPLA2- and 0.25 mg/kg rat anti-mouse CD25 Ab-treated arthritis group (*n* = 6); P60: 0.5 mg/kg bvPLA2- and 10 μg/kg P60-treated arthritis group (*n* = 6); NOR Ab: 0.5 mg/kg bvPLA2- and 0.25 mg/kg normal rat Ab-treated arthritis group (*n* = 6); SCRAMB: 0.5 mg/kg PLA2- and 10 μg/kg scrambled P60-treated arthritis group (*n* = 6). One-way ANOVA followed by Newman–Keuls post hoc test was used for comparisons of arthritic behaviors above among the groups. For comparisons between Anti-CD25 Ab and NOR Ab groups, and P60 and SCRAMB groups, paired Student’s t-test were performed. In all analyses, statistical differences were considered significant at* p* < 0.05. ^###^*p* < 0.001 vs. the NOR group, ^$$$^*p* < 0.001 versus the CIA group, and *p < 0.05, **p < 0.01, ***p < 0.001 versus the CIA_PLA2 group. ^$$^*p* < 0.01 (paired Student´s t-test within between Anti-CD25 Ab and NOR Ab groups), and ^&&&^*p* < 0.001 (paired Student´s t-test within between P60 and SCRAMB groups).

**Figure 6:**
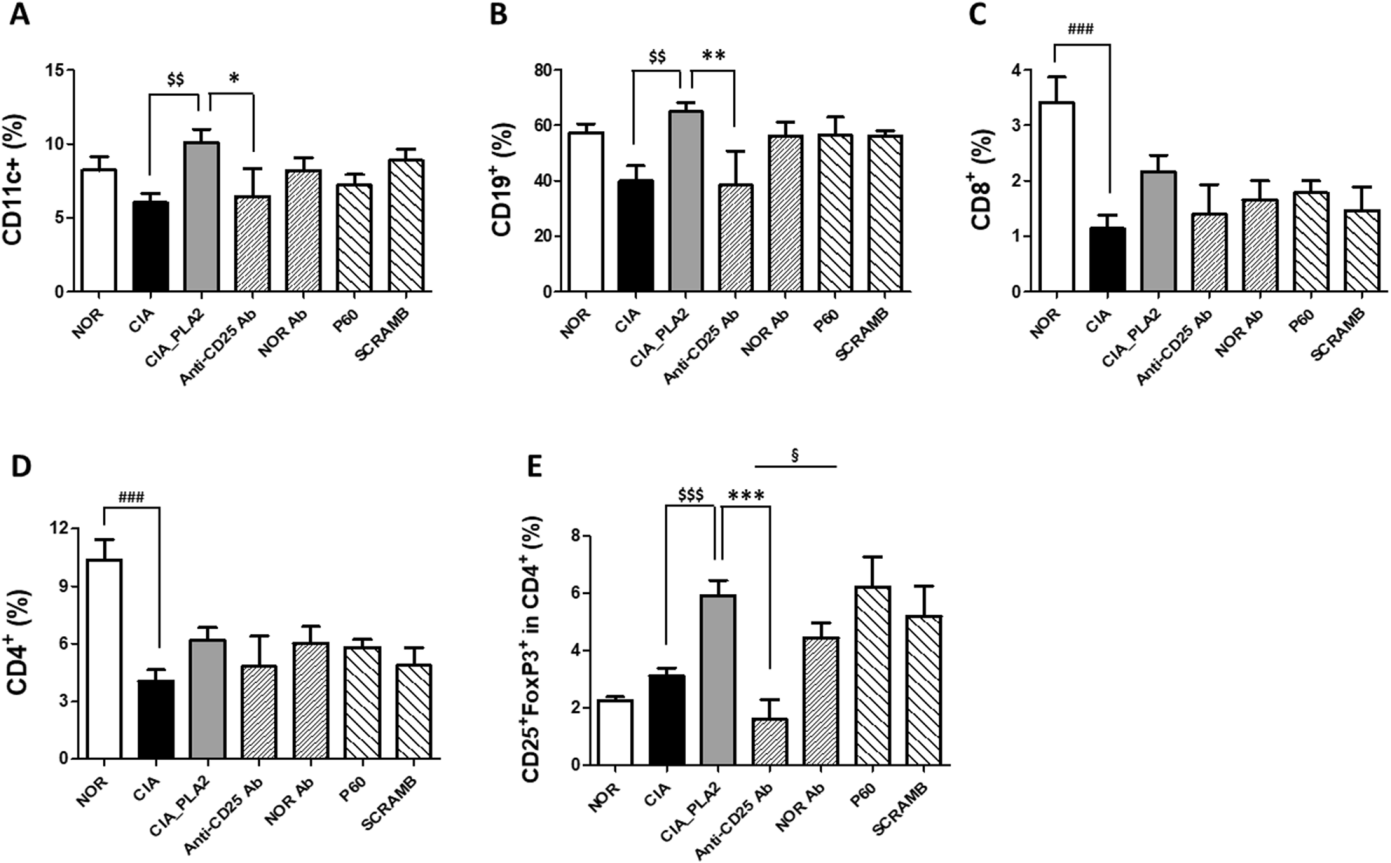
Flow cytometric analyses of dendritic cells (CD11c^+^, **A**), B cells (CD19^+^, **B**), cytotoxic T cells (CD8^+^, **C**), helper T cells (CD4^+^, **D**), and regulatory T cells (CD4^+^ CD25^+^ Foxp3^+^, **E**) in the spleens of DBA/1 mice with CIA. Three different spleens were randomly sampled in each group to analyze the T cell subsets. One-way ANOVA followed by Newman–Keuls post hoc test was used for comparisons of flow cytometric data among the groups. For comparisons between Anti-CD25 Ab and NOR Ab groups, and P60 and SCRAMB groups, paired Student’s t-test were performed. In all analyses, statistical differences were considered significant at* p* < 0.05. ^###^*p* < 0.001 versus the NOR group, ^$$^*p* < 0.01, ^$$$^*p* < 0.001 versus the CIA group, and *p < 0.05, **p < 0.01, ***p < 0.001 versus the CIA_PLA2 group. ^$$^*p* < 0.01 (paired Student´s t-test within between Anti-CD25 Ab and NOR Ab groups), and ^&&&^*P* < 0.001 (paired Student´s t-test within between P60 and SCRAMB groups).

All statistical analyses were performed using GraphPad Prism 5.0 (GraphPad Software Inc, San Diego, CA, USA). Data were expressed as the mean ± standard error of the mean (SEM). Body weight, squeaking score, paw volume, and arthritis index score in Fig. 2 were analyzed by two-way repeated-measures ANOVA, followed by Bonferroni’s post-hoc test. One-way ANOVA followed by Newman–Keuls post hoc test was used for comparisons of inflammation score, BMD and arthritic behaviors (Figs. 3, 4 and 5), and flow cytometric data (Fig. 6) among the groups. For comparisons between Anti-CD25 Ab and NOR Ab groups, and P60 and SCRAMB groups in Fig. 5 and 6, paired Student’s t-test were performed. In all analyses, statistical differences were considered significant at* p* < 0.05.

## Data Availability

The datasets generated during and/or analysed during the current study are available from the corresponding author on reasonable request.
